# Changes in balance performance and Its determining factors in the lower leg during the competition season in adolescent football players

**DOI:** 10.3389/fspor.2026.1769946

**Published:** 2026-05-28

**Authors:** Inese Pontaga, Agris Liepa, Valters Abolins, Andrew Rinaldi Sinulingga, Kristaps Slaidins

**Affiliations:** 1 Department of Sports Theory, Latvian Academy of Sports Education, Riga Stradins University, Riga, Latvia; 2 Health Care Research Center and the Sports Science Research Laboratory, Latvian Academy of Sports Education, Riga Stradins University, Riga, Latvia; 3 Institute of Occupational Safety and Environmental Health, Riga Stradins University, Riga, Latvia; 4 Department of Sport Education, University of Riau, Pekanbaru, Riau, Indonesia; 5 Department of Individual and Team Sports, Latvian Academy of Sports Education, Riga Stradins University, Riga, Latvia

**Keywords:** ankle range of movement, dynamic balance, kinesthetic sense, shin muscle strength, static balance

## Abstract

The aim of our study was to evaluate changes in static and dynamic balance, shin muscle strength, active and passive ankle range of movement (ROM), and foot kinesthetic acuity during the first 8 weeks of the competitive season in male adolescent football players. Thirteen players (age 15.26 ± 0.93 years, Tanner stages 3–4) participated. Static and dynamic single-leg balance and foot kinesthetic sense were assessed using a stabilometric platform. Ankle plantarflexion–dorsiflexion and inversion–eversion ROM were measured with a goniometer. Peak isometric force of the ankle plantar flexors, dorsiflexors, invertors, and evertors was measured with a handheld dynamometer, and peak torque was calculated as force × lever arm length. Eight weeks of competitive period was associated with static balance improvement: mean anterior-posterior and medial-lateral sway speeds decreased bilaterally (*P* = 0.024). Center of pressure (CoP) perimeter decreased significantly in the non-dominant (N) leg (*P* = 0.028). CoP perimeter and sway speed were positively correlated with passive dorsiflexion/plantarflexion ROM, and CoP ellipse area was correlated with active plantarflexion ROM (*P* ≤ 0.049). Dynamic balance improvement: the trunk total standard deviation (TTD) angle decreased when standing on the dominant (D) and N legs (*P* ≤ 0.018). TTD angle was negatively correlated with peak ankle dorsiflexion, eversion, and inversion torques (*P* ≤ 0.039). Passive plantarflexion ROM decreased bilaterally (P ≤ 0.04), whereas active ROM remained unchanged (*P* ≥ 0.05). Absolute and relative peak isometric dorsiflexion torques increased bilaterally (*P* ≤ 0.003), but side asymmetry (higher plantar- and dorsiflexion torques in the D leg) persisted (*P* ≤ 0.045). Mean kinesthetic sense error was lower with the D leg, whereas trial completion time was shorter with the N leg in both sessions (*P* ≤ 0.032).

## 1 Introduction

Balance is crucial for everyday activities such as standing and walking without support and for reducing the likelihood of falls and related injuries (1). In addition, improving dynamic balance can play an important role in fall prevention, particularly in response to unexpected external perturbations (1). Balance is the limiting factor in athletic performance: when balance is impaired, sports performance declines and/or the frequency of falls and injury risk increases (2).

Balance performance (functional balance, postural balance, or balance ability) refers to the ability to maintain physical equilibrium and stability during various postures or activities (3). It can be divided into static and dynamic balance (4). Macpherson and Horak (5) defined static balance as the ability to maintain equilibrium in undisturbed environments, such as standing still. In contrast, dynamic balance refers to the capacity to perform tasks while maintaining or regaining a stable position or to preserve balance on unstable surfaces with minimal extraneous movement (3, 6). The results reported by Pau et al. (7) and Meiners & Loudon (8) demonstrated that static and dynamic balance characteristics were not associated with one another. Therefore, the assessment of balance in football players should be performed using both dynamic and static tests. Similarly, in our prior work, we found no significant relationship between static and dynamic balance in young football players (9) and in adult athletes trained in team sports—basketball, volleyball, and football players (10). Evidence suggests that dynamic balance is a key determinant of overall performance in football players (11, 12).

Balance performance, particularly in a one-leg stance, is crucial for enhancing a football player's ability to perform high-intensity explosive actions, such as sprints, jumps, accelerations, and decelerations during changes of direction—key physical capacities required for success in competition (12, 13). Paillard et al. (14) observed that national-level football players possess better static and dynamic balance, as well as different strategies to maintain it in a one-leg stance, than regional-level players. Therefore, in football-specific conditions, balance can be considered a criterion of the player's performance level. For instance, Scinicarelli et al. (15) observed a strong correlation between single-dominant-leg dynamic balance performance and multi-directional speed performance on the lower extremity functional test in football players. Mitrousis et al. (16) reported that an 8-week balance training intervention improved both static and dynamic balance and increased shooting accuracy in the dominant (D) leg of 12- to 13-year-old football players. Similarly, Cè et al. (17) determined low to moderate correlations between the technical proficiency of 11-year-old football players and their single-leg balance performance, with the strongest associations observed in the N leg (r = 0.30–0.48). Significant correlations were observed between balance in a one-leg stance and kicking accuracy, but not speed, in male and female undergraduate high school students possessing an intermediate level of football skills: balance on the left leg showed a strong correlation with the kicking accuracy of the right (D) leg; in contrast, this pattern of relationships did not appear between single-leg balance and kicking accuracy of the left (N) leg (18). These results provide preliminary support for the importance of balance ability in kicking performance (18).

Nevertheless, studies have demonstrated that regularly performing balance training exercises can significantly lower the incidence of sports-related injuries among adolescent athletes (19). Moreover, to more effectively improve balance and lower the risk of injury, it is recommended to incorporate both balance exercises combined with muscle-strengthening activities (20) and muscle-strengthening exercises paired with flexibility training (21).

Static and dynamic balance depends on (1) leg and core muscle strength in the population of young healthy adults (22, 23), football players (11, 24), and futsal players (25); (2) an active and passive range of movement (ROM) in the ankle, knee, and hip joints (22, 25), which is related to connective tissue passive and active (leg muscle tone and co-contraction) stiffness (19, 26–28); and (3) kinesthetic sense acuity (proprioception and skin tactile senses) of the feet (29–32).

The effects of the pubertal growth spurt on balance abilities and the accuracy of kinesthetic perception remain insufficiently understood in adolescent football players (33, 34). Researchers have indicated that adolescents undergoing rapid growth—exceeding 0.6 cm per month—experience decreased coordination, poorer balance (often manifesting as awkwardness), and reduced kinesthetic sensitivity (35–37). This has been linked to slower processing of motor commands in the adolescent brain (36), resulting in less tightly controlled movements and a lack of automatization in balance control and interlimb/intersegmental coordination (38). Conversely, some studies have found no decline in motor skills among adolescent athletes (39, 40).

There is limited research on how various factors of the lower leg influence static and dynamic balance performance in growing adolescent football players, as well as the interactions among these factors. For example, Mitrousis et al. (16) investigated the effects of 8 weeks of balance training on static and dynamic balance, as well as technical skills in adolescent football players. They observed improvements in both balance and shooting accuracy with the dominant leg; however, they did not assess shin muscle strength or ankle ROM. Conversely, Cug et al. (41) noted that a four-week balance training program enhanced dynamic balance and the force production of the ankle's plantar flexor, dorsiflexor, invertor, and evertor muscle groups in healthy young adults, but not in football players. The influence of regular training combined with weekend match play on static and dynamic balance, peak shin muscle strength, active and passive ROM in the subtalar and talocrural joints, and the kinesthetic sense acuity of the feet during the competitive season in growing adolescent footballers remains under-researched on a large scale. Additionally, the training surfaces change with the season, ranging from the hard floor of the sports hall in the preparatory period to natural or artificial turf during the competition period, each of which demands specific adaptations in balance performance (42).

The research hypothesis proposes that static and dynamic balance performance, shin muscle strength, active and passive ROM in the talocrural and subtalar joints, and the accuracy of foot kinesthetic sense would improve during the first 8 weeks of the competition season (spring) in male adolescent football players.

The aim of our study was to evaluate changes in static and dynamic balance, shin muscle strength, active and passive ankle range of movement (ROM) in the subtalar and talocrural joints, and foot kinesthetic acuity, as well as correlations among these characteristics during the first 8 weeks of the competitive season in male adolescent football players.

## 2 Material and methods

### 2.1 Study design and participants

The research design was a longitudinal observational study to evaluate changes in the selected characteristics during the first 8 weeks of the competition period of one macrocycle in adolescent football players. The study took place in spring, during the transition from the firm indoor flooring used in the preparation period to the artificial grass surface of the outdoor stadium used in the competition period. The outside air temperature varied from +6°C to +23°C in April and May, and the humidity ranged from 45% to 90% on different days.

We investigated football players who attended training sessions at the same club, “Football Park”. The selected participants were players aged 14 to 16 years, classified as Tanner stages 3 and 4. They were all from middle-income families. The initial number of players was 20. Based on exclusion criteria, 3 players were dropped from the study due to health conditions—one because of a lower extremity injury in the previous 6 months and two due to upper respiratory infections. Four football players were not evaluated at the 8-week follow-up; three sustained injuries before the second assessment, and one transferred to another club. Therefore, the analysis was conducted using data from only 13 players.

Thirteen male amateur football players, aged 15.26 ± 0.93 years, from the football club “Football Park” participated. Their mean football training experience was 8.1 ± 2.6 years. The football players attended regular training sessions five times per week (8.4 ± 2.1 training hours per week) and participated in competitions on weekends. The sports physician estimated the maturation of the adolescent players using the Tanner scale (43); only participants classified as Tanner stages 3–4 were included in the investigation.

Exclusion criteria for participants were:

(1) pain in the lower limbs during testing, a lower extremity or back injury within the previous six months, or surgery within the last 12 months, as these conditions may impair kinesthetic or somatosensory function (44);
(2) vestibular disorders, or ongoing treatment for inner ear, sinus, or upper respiratory tract infections, which can reduce vestibular sensitivity and postural stability (45);
(3) concussion within the three months preceding the study, which can alter central nervous system processing of sensory inputs, a key prerequisite for balance (46);
(4) a positive Romberg test with eyes closed, defined as loss of balance, compensatory stepping, or falls (47); and
(5) marked deviations from normative lower-limb alignment, including genu varum or genu valgum (femorotibial angle > 7°) (48), femoral anteversion > 15° or femoral retroversion < 8° (49), calcaneal inversion or eversion in the frontal plane, viewed from behind, >5° (50), or anatomical leg-length discrepancy ≥ 0.01 m (51).

The physiotherapist evaluated the anatomical characteristics of the lower limbs in the frontal, sagittal, and horizontal planes and excluded football players exhibiting deviations from normative alignment, as defined by the criteria above.

Exclusion-related information was obtained through anamnesis by interviewing participants, without the use of a standardized written questionnaire. The Romberg test with eyes closed was negative in all included participants.

The players identified their preferred or dominant (D) and non-preferred or non-dominant (N) legs using the “Waterloo Footedness Questionnaire-Revised” (WFQ-R) (52).

### 2.2 Data collection

The participants, their parents, and coaches were informed about the study's experimental procedures, risks, and benefits. The parents voluntarily signed the informed consent form, allowing their sons to participate in the study. The study was conducted in accordance with the principles outlined in the Declaration of Helsinki and was approved by the Ethics Committee of Riga Stradins University (Decision No. 2-PĒK-4/890/2025, July 28, 2025).

The assessment of football players was conducted at the Health Care Research Center and the Sports Science Research Laboratory of the Latvian Academy of Sports Education, Riga Stradins University, one to two hours before the routine training session. The footballers did not participate in training sessions or matches 48 h prior to the laboratory testing. The laboratory air temperature was +20°C ± 2°C, and the humidity ranged from 45% to 50%.

Before measurements, participants performed general warm-up exercises, local warm-up, and active stretching for the legs and trunk for 10 min.

Before formal testing, each participant completed three practice trials of each test (static and dynamic balance, acuity of kinesthetic sense, and ankle flexibility measurements) with both the dominant and non-dominant legs to familiarize themselves with the equipment. The sequence of legs in the tests was randomized.

### 2.3 Anthropometry

Participants' heights were measured (cm) using an MZ10020 ultrasound height measuring unit (ADE, Hamburg). The body mass of the participants wearing sports briefs was measured in kilograms (kg) using a BC-418 body composition analyzer (Tanita Corporation, Japan). The measurements were repeated twice: at the beginning of the competition season and after eight weeks to assess the growth rate of the adolescent participants. A body mass index (BMI) was calculated as BMI = a participant's body mass (kg)/ height (m)^2^ (53).

### 2.4 Balance performance measurement

Static and dynamic balance, as well as kinesthetic sense accuracy, were assessed using the ProKin 252 stabilometric platform (TecnoBody, Dalmine, Italy). The TecnoBody Prokin device has been employed not only to evaluate individuals with neurological and musculoskeletal conditions, but also within sports science, for testing dynamic and static balance (54). This is a valid and highly reliable computerized posturography system for assessing static and dynamic balance with moderate validity and moderate-to-excellent test-retest reliability (ICC 0.55–0.93) in young healthy sportive adults evaluated 3 times with 4–7 days intervals (55). All evaluations were performed by the same experienced physiotherapist, who had received training in balance assessment using the ProKin 252 stabilometric platform and had worked in the laboratory for several years. Inter-rater reliability was assessed using the intraclass correlation coefficient (ICC), and the physiotherapist achieved an ICC of at least 0.85 before starting data collection (56).

**Figure 1:**
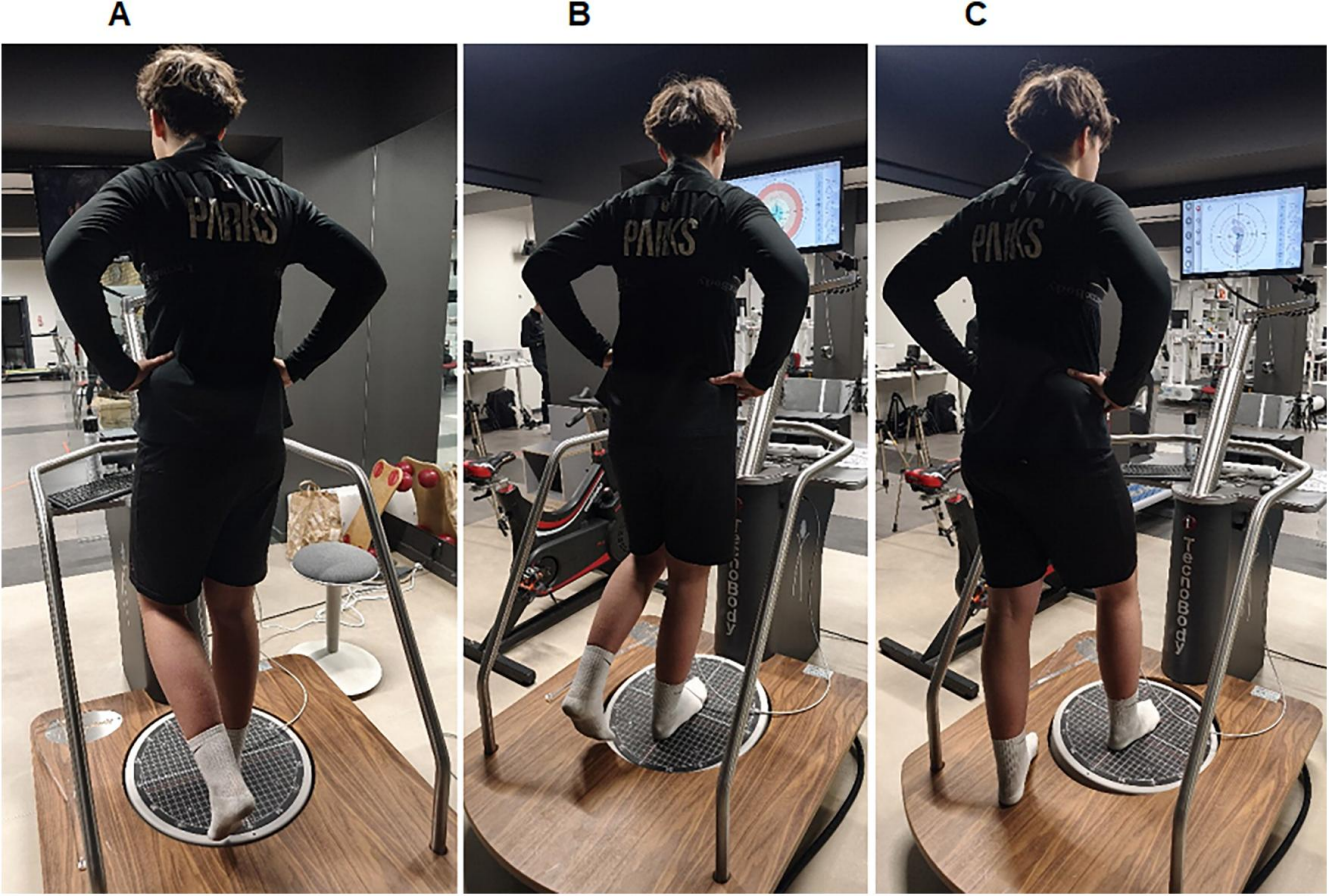
Static (**A**) and dynamic (**B**) balance assessment in a one-leg stance, the opposite leg was flexed at the knee, and kinesthetic sense accuracy (**C**) measurement (the participant positioned one leg on the stable surface of the balance platform, the tested leg was placed on the unstable balance platform) using the ProKin 252 stabilometric platform (TecnoBody, Dalmine, Italy).

The trunk sensor was positioned on the chest at the distal portion of the sternum (xiphoid process region). Balance characteristics were evaluated in a one-leg stance. The longitudinal axis of the leg was determined by drawing a tangent line from the midpoint of the middle toe to the central point of the heel. The participant positioned the middle of the foot over the center of the balance platform, placed both hands on the hips, and kept the opposite leg flexed at 45° at the knee joint throughout the test. No contact between the lifted leg and the stance leg was permitted (55); Figure 1A.

The static balance assessment. Subjects were instructed to maintain balance on one foot for 30 s with their eyes open and to focus on a stationary target. The same was repeated on the other leg. Following the completion of both tests, the device exhibited the following characteristics: ellipse area (EA) of the body's center of pressure (CoP) movements, perimeter (P) of the CoP movement area, mean anterior-posterior sway speed (APS), and mean medial-lateral sway speed (MLS). The perimeter of the body sway area (P) was calculated as the total length of the irregular CoP sway trajectory. The body's CoP sway area (EA) was an established elliptical form that covered at least 90% or 95% of the chaotic sway lines (55).

The dynamic balance assessment. The players were instructed to maintain balance in a one-leg stance, while looking straight ahead at a screen for 30 s with their eyes open. The object (a cross) on the screen, which corresponded to the platform sway variability, had to be maintained at the center of the visual target (also presented on the screen). The system's movable balance platform was driven by air piston servo motors and conducted measurements in every direction within an operating angle of 15°; Figure 1B. The device presented data on the characteristics of dynamic balance, including the total stability index (TSI) and total trunk standard deviation angle (TTD). TSI was classified as normative for trained subjects with a value of 0–0.83, normal between 0.84 and 2.32, and poor dynamic balance if it exceeded 2.33 (9).

### 2.5 Kinesthetic sense acuity measurement

The ProKin 252 stabilometric platform is designed to measure foot kinesthetic sense acuity (active movement trajectory repetition error) with high accuracy, aiding in rehabilitation for orthopaedical and neurological conditions (57). This device is among the most used systems for lower-limb rehabilitation. The ProKin 252 system offers a highly specialized, objective, computer-based approach to evaluating proprioceptive sense acuity of active movement trajectory repetition, in contrast to conventional functional testing methods (57). However, this is a general evaluation of kinesthetic sense and is not specifically tailored to football players. Research indicates that manual joint position matching—a form of functional assessment—exhibits greater variability and lower accuracy (±44%–75% accuracy differences) compared to objective, technology-based measurement approaches (58).

The foot kinesthetic sense acuity, or proprioception, was measured for each player's foot. A participant positioned one foot on a stable surface of the balance platform and looked straight ahead at the screen, where a circular ring was displayed. The opposite leg was placed on an unstable balance platform. The participant was instructed to follow the circular ring on the screen as accurately and quickly as possible with the tested (non-support) foot on the movable balance platform. The drawing process was performed on a movable platform in a clockwise direction using the right foot. The test was considered complete if three rings were drawn by the foot in less than one minute. The same task was repeated counterclockwise with the left foot; Figure 1C. The average trace error (ATE) score in percentage (%) and the trial time in seconds (s) were recorded for each foot.

ATE between 0% and 35% was classified as particularly good, values from 35 to 100% were regarded as adequate, and values exceeding 100% were interpreted as indicative of impaired proprioceptive control (59).

### 2.6 Range of motion measurement

The range of movement measured in ankle plantar and dorsal flexion of the talocrural joint, as well as ankle inversion and eversion of the subtalar joint, was assessed during both active and passive motions.

**Figure 2:**
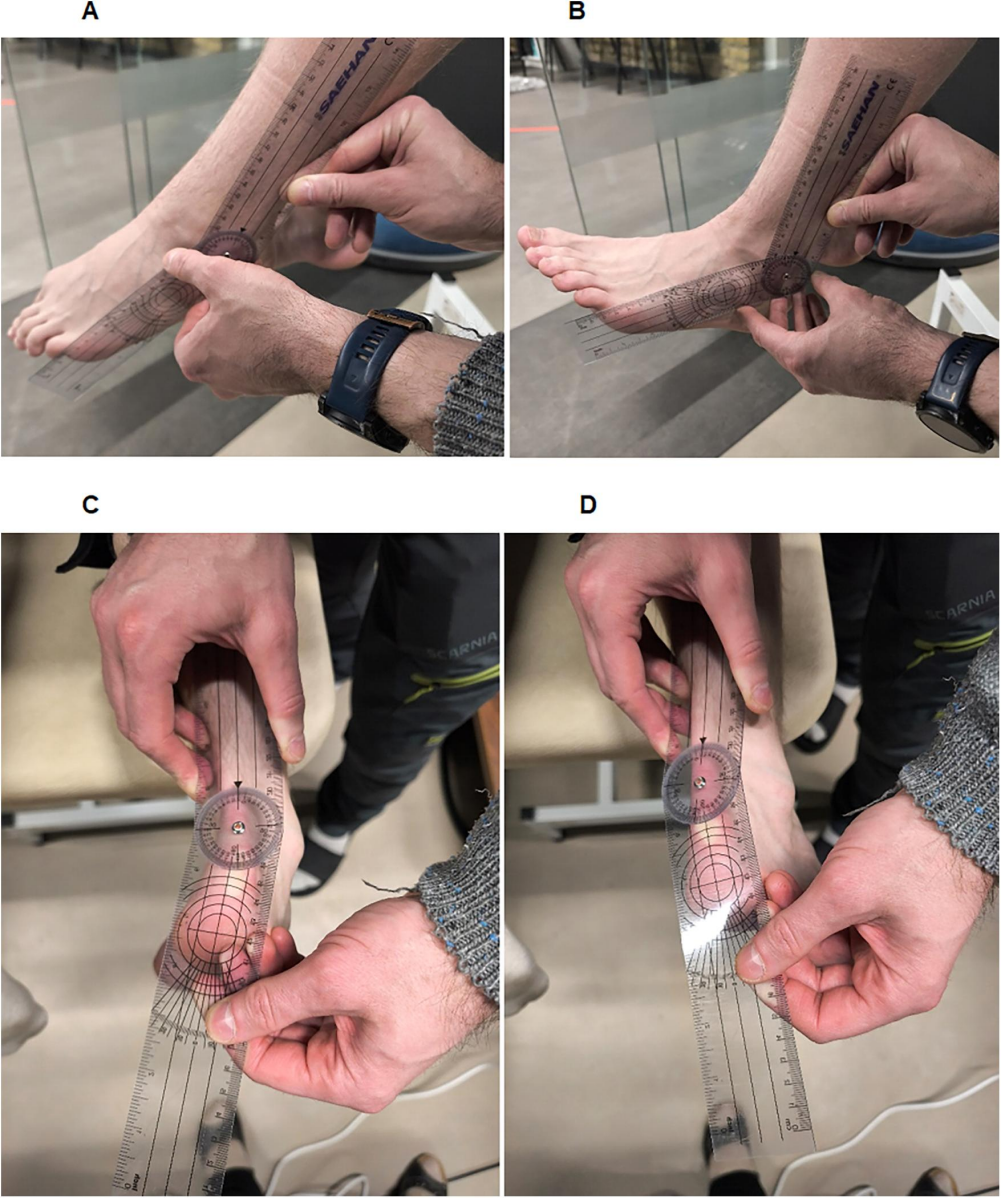
The range of movement (ROM) measurement in ankle plantar **(A)** and dorsal **(B)** flexion of the talocrural joint, and in ankle inversion **(C)** and eversion **(D)** of the subtalar joint.

To measure the range of movements (ROM) of the talocrural joint of the ankle, plantar flexion (PF) and dorsiflexion (DF) were assessed with participants seated and the knee flexed to an angle of 90°. The ROM was measured using a SAEHAN SH5205 goniometer (SAEHAN Ltd., Korea), following the protocols in “A Guide to Goniometry by Norkin and White” (60). Landmark: a dot was marked 2 cm below the tip of the lateral malleolus, and another dot was marked 10 cm above the tip of the lateral malleolus on the posterior border of the fibula. The goniometer's pivot point (fulcrum) was placed on the midpoint of the lateral malleolus, and the fixed arm was kept parallel to the lateral midline of the fibula. The movable arm of the goniometer followed the lateral midline of the fifth metatarsal bone; Figures 2A,B. Subtalar joint ROM measurement (inversion (IV) and eversion (EV): participants were placed in a prone position with the knee joint in full extension. Landmark: the midpoint between the malleoli on the posterior aspect of the ankle; the midline on the posterior aspect of the lower leg; and the midline of the posterior aspect of the calcaneus. The fulcrum of the goniometer was placed on the posterior midline between the lateral and medial aspects of the foot at the level of the metatarsal heads; the fixed arm was parallel to the lateral midline of the leg, and the movable arm was parallel to the posterior midline of the calcaneus (60); Figures 2C,D. The average score from three trials, measured by the same physiotherapist, was calculated.

### 2.7 Measurement of peak isometric strength in the shin muscles

The peak isometric force of the ankle dorsal and plantar flexor muscles, ankle invertor, and evertor muscles was measured using a handheld dynamometer (MicroFET2 Wireless; Hoggan Health Industries, West Jordan, USA) in Newtons (N). The primary contributor to error in ankle strength assessments is angular displacement in both the sagittal and horizontal planes, especially during plantarflexion trials (61). This indicates that precise control of the patient's limb position and movement is crucial for obtaining accurate measurements. Participants were positioned supine with knees straight, hands clasped, the non-tested leg in a hook-lying posture, and the tested foot hanging unsupported over the edge of the bed with the ankle in a neutral position. The same strong male physiotherapist performed all ankle muscle strength measurements. He stood upright, held the dynamometer in his dominant hand, and pushed it against the force exerted by the participant's muscles. The lower limb was stabilized by his other hand proximal to the ankle joint.

Before the strength measurements, two practice trials were performed to familiarize the participants with the testing procedures: one isometric sub-maximal strength trial and one maximal strength trial were administered as a warm-up before the start of the tests (11).

**Figure 3:**
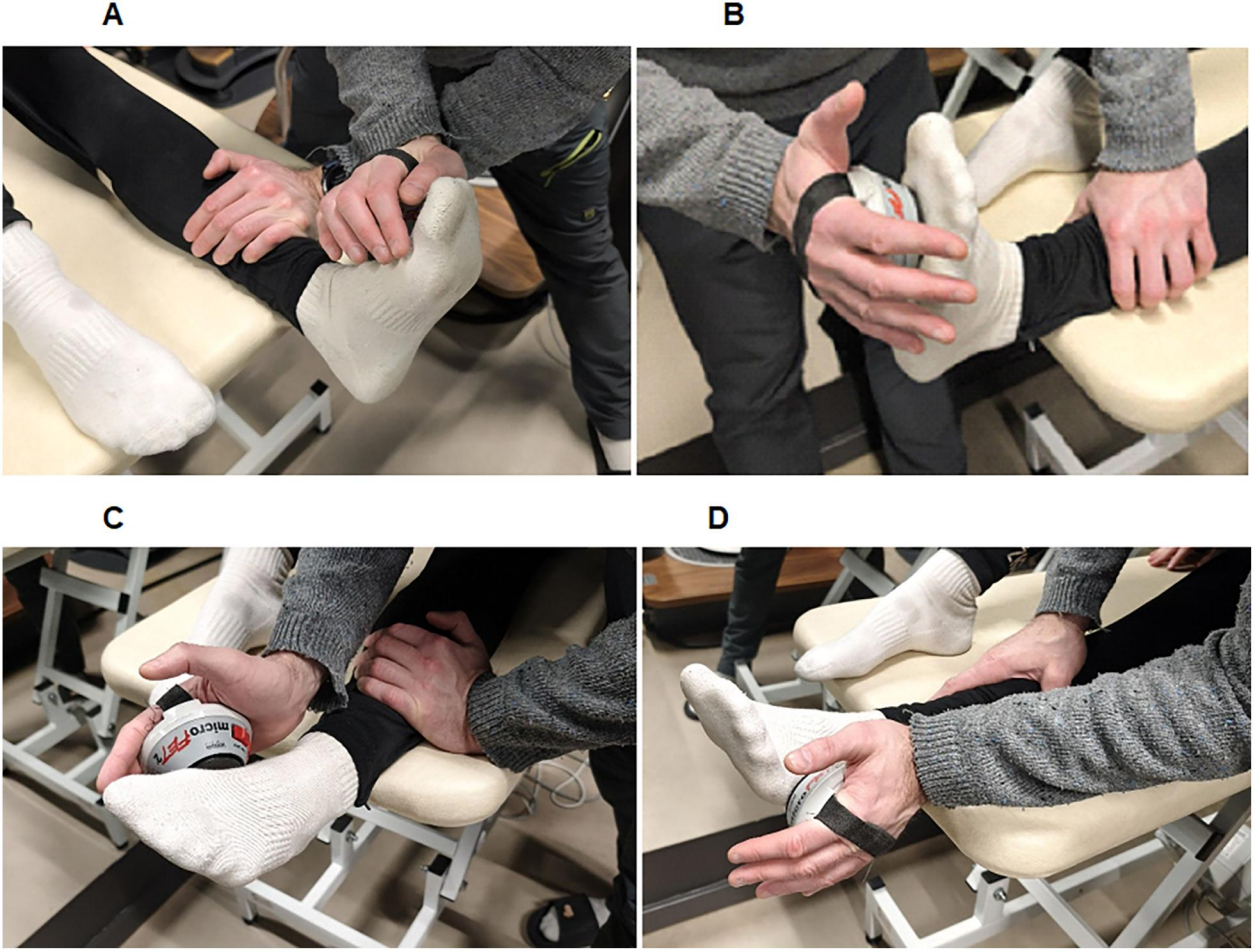
The peak isometric force of the ankle dorsal **(A)** and plantar flexor **(B)** muscles, ankle invertor **(C)**, and evertor **(D)** muscles, measured using a handheld dynamometer (MicroFET2 wireless; hoggan health industries, West Jordan, USA).

To measure the peak isometric force of the ankle dorsal flexor muscle, the dynamometer was positioned against the dorsal surface of the foot just proximal to the heads of the metatarsal bones (Figure 3A); to examine the plantar flexor muscle, it was placed on the plantar surface of the foot just proximal to the head of the first metatarsal bone (Figure 3B); to assess the ankle invertor muscle, it was positioned on the medial border of the foot at the midpoint of the shaft of the first metatarsal bone (Figure 3C); and to test the evertor muscle, it was placed on the lateral border of the foot at the midpoint of the fifth metatarsal bone (Figure 3D) (62). The dynamometer was pushed against the physiotherapist's hand as hard as possible for 3 s with each foot, with 3 repetitions at 60 s intervals. The maximum muscle-generated force depends on the participant's voluntary effort. Therefore, we encouraged each participant to push the dynamometer as strongly as possible. Three trials assessed the maximum force. The highest muscle-generated force measurement in each muscle group was selected for further analysis because the peak force assessment error may be only underestimated but not overestimated. The order of the isometric force testing in the D and N legs was randomized.

The ankle dorsal and plantar flexor muscle peak torque values were calculated using the best repetition force multiplied by the distance from the medial malleolus to the head of the first metatarsal bone. The ankle invertor peak torque values were calculated using the best repetition force multiplied by the distance from the medial malleolus to the midpoint of the shaft of the first metatarsal bone. Still, the ankle evertor muscle peak torque values were calculated using the best repetition force multiplied by the distance from the lateral malleolus to the lateral border of the foot at the midpoint of the fifth metatarsal bone. Then, the torques were divided by body mass (Nm/kg) to obtain relative peak torques.

### 2.8 Statistical analysis

The sample size was estimated using the G*Power program (version 3.1.9.7) for the present study. An effect size (f) of 0.6 was assumed, with a statistical power of 95% (β = 0.95) and a significance level of α = 0.05. These parameters were determined based on the results of a study that examined dynamic balance (63). The analysis indicated a minimum requirement of 12 participants per group.

The normality of the data distribution of the anthropometric characteristics, ankle passive and active range of movement (ROM), peak isometric shin muscle force, static and dynamic balance, and kinesthetic sense acuity characteristics were established by the Shapiro–Wilk test, and sphericity was evaluated with Mauchly's test. In cases where normality was not met, data were log-transformed (Supplementary Table S1). To test our hypotheses, analysis of variance (ANOVA) with repeated measures and pairwise comparisons with Bonferroni corrections was used. In cases where normality was not met even after log-transformation, Friedman's test and pairwise comparisons with the Wilcoxon signed-rank test were used. We examined the effects of Leg dominance (Dominant and Non-dominant) and Time (at the beginning of the competition season and after eight weeks) on each leg's characteristics. Correlations between leg characteristics were calculated using either Pearson's or Spearman's correlation, depending on the normality of the data. Statistical significance was set at *P* < 0.05. All statistical analyses were conducted using R version 4.4.3 (64).

## 3 Results

During the competition season, the number of training hours per week increased significantly, from 8.32 ± 1.97 h to 9.82 ± 1.97 h (*P* *=* *0.012*). The increase in mean height (+0.8 cm) and body mass (+1.02 kg) of young football players was statistically significant (*P* *<* *0.001*), but the BMI remained unchanged during the first eight weeks of the competition season. The anthropometric characteristics are shown in Table 1.

**Table 1:** Anthropometric characteristics of the adolescent football players (mean ± SD).

Time of measurements	A (years)	h (cm)	m (kg)	BMI (kg/m^2^)	TH (h)	TE (years)
Competition season beginning	15.26 ± 0.93	174.58 ± 6.49	64.32 ± 8.12	21.01 ± 2.67	8.32 ± 1.97	8.00 ± 2.58
8 weeks later	15.38 ± 0.93	175.38** ± 6.50	65,34** ± 7.79	21.26 ± 2.44	9.82* ± 1.97	8.12 ± 2.58
*P-value*	*0*.*347*	*<0*.*001*	*<0*.*001*	*0*.*051*	*0*.*012*	*0*.*443*

A, mean age; h, mean height; m, mean body mass; BMI, mean body mass index; TH, training hours per week; TE, training experience.

* *P* *<* *0.05*.

** *P* *<* *0.001*.

**Figure 4:**
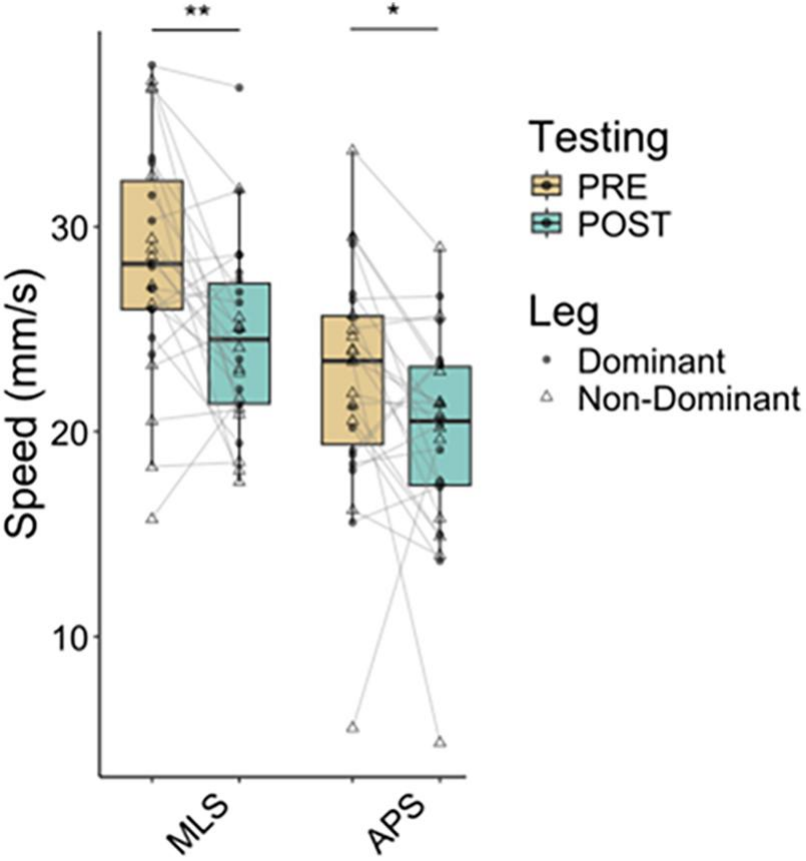
Static balance characteristics: mean medial-lateral sway speed (MLS) and mean anterior-posterior sway speed (APS) at the beginning of the competition season (PRE) and after 8 weeks spent in the competition season (POST). Box-and-whisker plots are shown, including all the individual data points. Solid grey lines connect the data points that belong to individual participants. The dominant leg data are shown as a filled circle, and the non-dominant leg data are shown as an empty triangle (* P < 0.05; ** P < 0.01).

At the beginning of the competition season and after eight weeks, all static balance characteristics did not differ significantly between the dominant (D) and non-dominant (N) leg stances (all *P* *>* *0.05*). Two-way ANOVA with repeated measures (RM) on the ellipse area showed no effect of testing time (*F1,12* *=* *1.90, P* *=* *0.193, η*^2^ *=* *0.037*), no effect of leg (*F1,12* *=* *2.57, P* *=* *0.135, η*^2^ *=* *0.062*), and no interaction between testing time and leg (*F1,12* *=* *0.18, P* *=* *0.681, η*^2^ *=* *0.003*). However, two-way ANOVA with RM on medial-lateral sway speed (MLS) showed a significant effect of testing time (*F1,12* *=* *10.79, P* *=* *0.007, η*^2^ *=* *0.131*), but no effect of leg (*F1,12* *=* *2.40, P* *=* *0.148, η*^2^ *=* *0.065*) and no interaction between testing time and leg (*F1,12* *=* *1.52, P* *=* *0.241, η*^2^ *=* *0.018*). Pairwise contrasts with Bonferroni corrections confirmed that after eight weeks of the competition period, MLS decreased (from 28.38 ± 5.62 mm/s to 24.63 ± 4.59 mm/s, *P* *=* *0.002*; see Figure 4). Similarly, there was a significant decrease in mean anterior-posterior sway speed (APS; from 22.71 ± 6.67 mm/s to 19.08 ± 5.99 mm/s; see Figure 4), confirmed with ANOVA (F1,12 = 6.62, *P* *=* *0.024*, *η*^2^ = 0.067) and pairwise corrections (*P* *=* *0.03*). We also observed a decrease in the perimeter (P) of the center of pressure movement area after the competition period in the N leg (from 1,217.28 ± 259.79 mm to 988.89 ± 113.78 mm), which was confirmed with Friedman's test [(3) = 14.077; *P* *=* *0.003*], followed by pairwise comparisons using the Wilcoxon signed-rank test (*P* *=* *0.028*).

**Figure 5:**
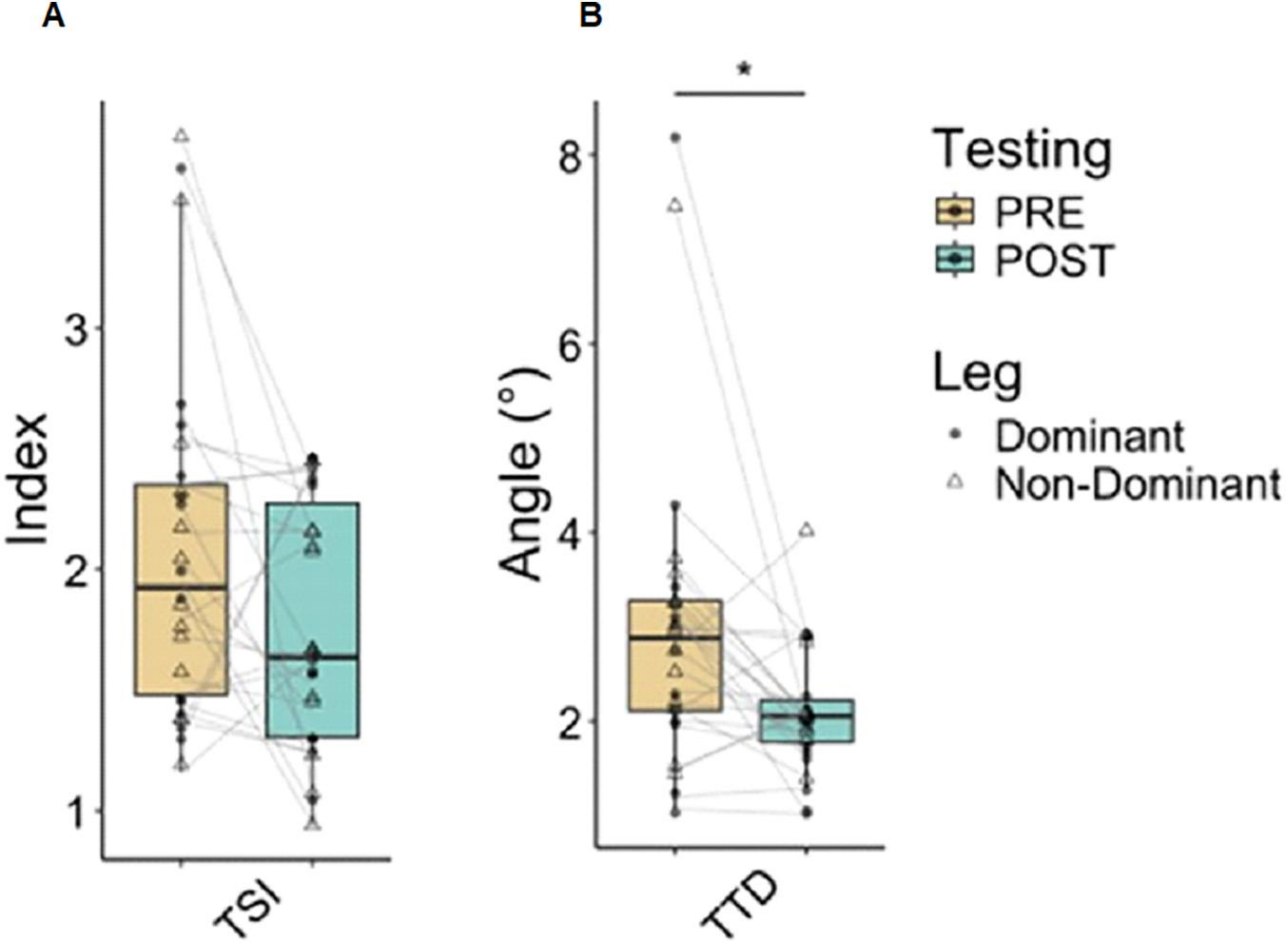
(**A**) Dynamic balance total stability index (TSI) and (**B**) trunk total standard deviation angle (TTD) at the beginning of the competition season (PRE) and after 8 weeks spent in the competition season (POST). Box-and-whisker plots are shown, including all individual data points. Solid grey lines connect the data points that belong to individual participants. The dominant leg is indicated as a filled circle, the non-dominant leg is shown as an empty triangle (* P < 0.05; ** P < 0.01).

At the beginning and after 8 weeks of the competition season, the dynamic balance total stability index (TSI) and trunk total standard deviation angle (TTD) did not differ significantly between the dominant (D) and non-dominant (N) leg stances (all *P* *>* *0.05*). The TSI was classified as normal for untrained subjects but too low for athletes (it ranged from 0.83 to 2.26) and did not change significantly over the season (*P* *>* *0.05*; see Figure 5A). However, after eight weeks of the competition season, the TTD angle enhanced (decreased) significantly (from 3.02° ± 1.6° to 2.11° ± 0.7°; see Figure 5B; standing on the N leg and from 2.97° ± 1.8° to 1.96° ± 0.67° while standing on the D leg). This was confirmed with a two-way ANOVA with RM (*F1,12* *=* *7.43, P* *=* *0.018, η*^2^ *=* *0.128*).

There were no significant differences in passive ankle joint ROM between the two limbs at the beginning of the competition season and after 8 weeks during the competitive season (all *P* *>* *0.05*). The passive plantar flexion ROM significantly reduced in both ankles after 8 weeks of the competition season, from 63° ± 6° to 53° ± 6° in the D leg, the difference was 10° (*P* *=* *0.040*) and from 63° ± 7° to 53° ± 6° in the N leg, the difference was 10° (*P* *=* *0.025*), which was confirmed with Friedman's test and Pairwise comparisons using the Wilcoxon signed rank test (Table 2).

**Table 2:** Passive and active range of movement (mean ± SD) in degrees (°) in the dominant and non-dominant ankle joint in adolescent football players.

Ankle joint's ROM	Leg	Competition season beginning	Competition season, 8 weeks later	*P-value*
Passive plantar flexion	D	60 ± 7	54* ± 6	0.0076
	N	61 ± 6	53** ± 5	0.0002
*P-value*		>0*.*05	>0*.*05	
Passive dorsal flexion	D	15 ± 5	16 ± 7	>0.05
	N	16 ± 6	16 ± 6	>0.05
*P-value*		>0*.*05	>0*.*05	
Passive inversion	D	19 ± 5	21 ± 3	>0.05
	N	20 ± 4	21 ± 3	>0.05
*P-value*		>0*.*05	>0*.*05	
Passive eversion	D	10 ± 3	10 ± 3	>0.05
	N	11 ± 4	11 ± 3	>0.05
*P-value*		>0*.*05	>0*.*05	
Active plantar flexion	D	42 ± 6	46 ± 6	>0.05
	N	43 ± 7	46 ± 5	>0.05
*P-value*		>0*.*05	>0*.*05	
Active dorsal flexion	D	10 ± 3	10 ± 7	>0.05
	N	11 ± 4	10 ± 6	>0.05
*P-value*		>0*.*05	>0*.*05	
Active inversion	D	12 ± 2	11 ± 3	>0.05
	N	12 ± 2	10 ± 2	>0.05
*P-value*		>0*.*05	>0*.*05	
Active eversion	D	5 ± 2	5 ± 2	>0.05
	N	6 ± 2	5 ± 2	>0.05
*P-value*		>0*.*05	>0*.*05	

ROM, range of movement; D, dominant leg; N, non-dominant leg.

* *P* < 0.01.

** *P* < 0.001.

The active ROM of the ankle joint in plantar and dorsal flexion, inversion, and eversion did not differ significantly between the limbs at the beginning of the competition season and after 8 weeks during the competitive season (*P* *>* *0.05*). After 8 weeks spent in the competition season, the active ankle ROMs did not change (*P* *>* *0.05*); the data are shown in Table 2.

**Figure 6:**
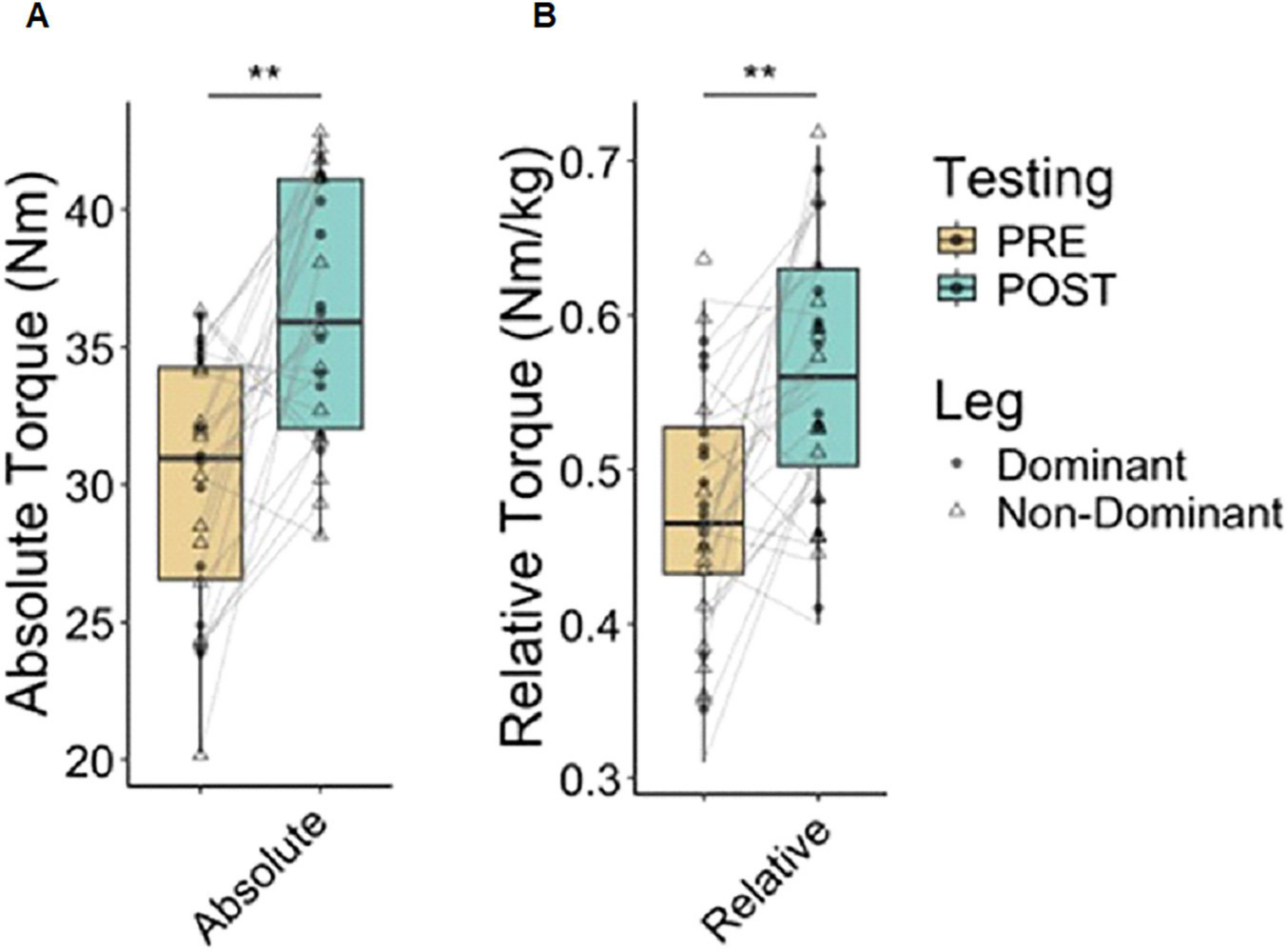
(**A**) Absolute peak torque in dorsiflexion and (**B**) relative peak torque in dorsiflexion at the beginning of the competition season (PRE) and after 8 weeks spent in the competition season (POST). Box-and-whisker plots are shown, including all individual data points. Solid grey lines connect the data points that belong to individual participants. The dominant leg is indicated as a filled circle, while the non-dominant leg is shown as an empty triangle (* P < 0.05; ** P < 0.01).

The absolute isometric peak torques of ankle plantar flexion were higher in the D leg—30.1 ± 10.0 Nm compared to the N leg—27.9 ± 9.2 Nm, the difference was 2.2 Nm (*F_1,12_* *=* *6.67, P* *=* *0.024, η*^2^ *=* *0.020*). Similarly, the absolute isometric peak torque side-asymmetry was detected for the ankle dorsiflexion, where the absolute peak torque was higher in the D leg—28.4 ± 7.1 Nm compared to the N leg—27.8 ± 7.2 Nm, the difference was 1.6 Nm (*F_1,12_* *=* *5.03, P* *=* *0.045, η*^2^ *=* *0.031*); this peak torque also increased from 30.1 ± 5.0 Nm to 36.3 ± 5.0 Nm in both legs over 8 weeks (*F_1,12_* *=* *14.41, P* *=* *0.003, η*^2^ *=* *0.328*; see Figure 6A). Additionally, the relative peak torque in dorsiflexion had lower values in the N leg—0.43 ± 0.09 Nm/kg than in the D leg—0.47 ± 0.08 Nm/kg, the difference was 0.04 Nm/kg (F_1,12_ = 5.40, *P* = 0.039, *η*^2^ = 0.026) and increased during the 8 weeks from 0.47 ± 0.08 Nm/kg to 0.56 ± 0.08 Nm/kg in both legs (*F_1,12_* *=* *12.48, P* *=* *0.004, η*^2^ *=* *0.257*; see Figure 6B). However, significant strength (absolute and relative peak torques) side asymmetry was not observed in the ankle invertor and evertor muscles (all *P* *>* *0.05*).

In the foot kinesthetic sense acuity assessment, the average tracking error score (ATE) was significantly smaller when the test was performed with the D leg (37% ± 12%) than with the N (41% ± 12%) (*P* *=* *0.021*), the difference was 4%, at the beginning of the competition season, and after 8 weeks spent in the competition season: 37% ± 9% in the D leg and 40% ± 9% in the N leg (*P* *=* *0.021*), the difference was smaller: 3%. The ATE was considered normal but not optimal, with values ranging from 35% to 100% (59).

The mean kinesthetic sense test trial time was shorter when performing the task with the N leg (30 ± 9 s) than with the D leg (37 ± 10 s) (*F_1,12_* *=* *7.01, P* *=* *0.021, η*^2^ *=* *0.033*) (the difference between the legs was 7 s), and it did not significantly change after 8 weeks of the competition period (*F_1,12_* *=* *0.004, P* *=* *0.949, η*^2^ *=* *0.0002*), when it was 23 ± 7 s for the N leg and 25 ± 8 s for the D leg (the difference between the means reduced to 2 s).

Significant positive correlations were determined between the passive ankle plantar flexion ROM and the characteristics of static balance: (1) the P of the body CoP movements (*R* *=* *0.433, P* *=* *0.001*); (2) the mean MLS speed (*R* *=* *0.384, P* *=* *0.005*); (3) the mean APS speed (*R* *=* *0.274, P* *=* *0.049*), Table 3.

**Table 3:** Statistically significant correlations between ankle parameters and balance characteristics.

Ankle variable	Balance variable	*r*	*P-value*
Passive plantar flexion ROM (°)	CoP P, mm	*0*.*433*	*0*.*001*
Passive plantar flexion ROM (°)	Mean MLS speed, mm/s	*0*.*384*	*0*.*005*
Passive plantar flexion ROM (°)	Mean APS speed, mm/s	*0*.*274*	*0*.*049*
Passive dorsiflexion ROM (°)	Mean APS speed, mm/s	*0*.*287*	*0*.*039*
Active plantar flexion ROM (°)	EA (CoP), mm2	*−0*.*293*	*0*.*035*
Relative dorsiflexion torque, Nm/kg	TTD angle (°)	*−0*.*386*	*0*.*005*
Relative eversion torque, Nm/kg	TTD angle (°)	*−0*.*337*	*0*.*015*
Relative inversion torque, Nm/kg	TTD angle (°)	*−0*.*287*	*0*.*039*

ROM, range of movement; CoP P, perimeter of body center of pressure sways; APS, mean anterior-posterior sway speed; MLS, mean medial-lateral sway speed; EA, ellipse area of CoP movement; TTD, trunk total standard deviation angle.

A significant positive correlation was found between passive ankle dorsiflexion ROM and the mean APS speed (*R* *=* *0.287, P* *=* *0.039*) during the static balance test (Table 3).

A negative correlation was observed between the active ankle plantar flexion ROM and the EA of the body's CoP movements (*R* = −0.293, *P* = 0.035) in the static balance test (Table 3).

A significant negative correlation was identified between relative ankle dorsal flexion torque and dynamic balance, characterized by the TTD angle (*R* *=* *−0.386, P* *=* *0.005*). The TTD angle was reduced as ankle dorsiflexor torque increased (Table 3).

A significant negative correlation was observed between relative ankle eversion torque and dynamic balance characterized by the TTD angle (*R* *=* *−0.337, P* *=* *0.015*). The TTD angle decreased as ankle eversion torque increased (Table 3).

A significant negative correlation was detected between the relative ankle inversion torque and the dynamic balance characteristic of the TTD angle (*R* *=* *−0.287, P* *=* *0.039*). The TTD angle declined as ankle inversion torque increased (Table 3).

## 4 Discussion

Despite the significant increase in the mean height (+0.8 cm) and body mass (+1.02 kg) of the participants, the 8-week competition period was associated with improvements in static balance characteristics during one-leg stances on both the D and N legs: the mean anterior-posterior (APS) and medial-lateral sway speeds (MLS) decreased, and the perimeter (P) of the body's center of pressure (CoP) significantly decreased only when standing on the N leg (*P* *≤* *0.024*).

The dynamic balance characteristics (TSI and TTD) while standing on the D and N legs were symmetrical at the beginning and after 8 weeks of the competition season (*P* ≥ 0.05). The competitive season led to improvements in dynamic balance in adolescent football players who are still developing, as shown by a reduction in overall trunk standard deviation (TTD) angles while standing on the D and N legs (*P* *≤* *0.018*).

Nevertheless, the mean CoP sway speed in both directions decreased when standing on the D and N legs; we observed that the perimeter (P) of the body's CoP significantly reduced only when standing on the N leg in adolescent footballers during the first 8 weeks of the competition period. This could be explained by the specialization of each leg of a football player in performing different tasks during the competition. Football players' motor skills or mobility tasks (passing, dribbling, receiving, and juggling the ball in the air) were performed mostly with the D leg. The N leg served a stabilizing function, providing the player with body support and helping maintain balance during the mobility tasks of the D leg (52). This coincided with a more pronounced improvement in static balance in the N or supporting leg of the footballer, associated with an increase in the hours regularly spent in football training sessions and playing matches. Improvement in static balance (reduction of the CoP sway area) was observed by Dafkou et al. (65) solely in the N leg due to the implementation of small doses of hamstring eccentric, proprioception, and core stability exercises into a routine training program for 8 weeks in adolescent (16–19-year-old) football players. Similarly, the group of young adult football players showed better static balance when standing on the left leg than the sedentary group (*P* *<* *0.05*) (66). Within both the basketball player and windsurfer groups, there were no significant differences in single-leg static balance performance between the D and N legs (66). Jadczak et al. (67) found that as the competitive level of adult football players increased, their static and dynamic balance improved, with professional players showing the highest performance and exhibiting superior body stability during play. They further found that professional footballers showed superior dynamic balance in the N leg compared with the D leg. In contrast to the previous study, other researchers found no significant difference in professional football players' ability to sustain dynamic balance when standing on one leg vs. the other (68). Therefore, the symmetry of balance performance could be associated with the intensity and volume of regularly executed asymmetrical exercises and could change during the competition season.

Based on our findings, the ranges of movement (ROM's) for ankle passive and active plantarflexion, dorsiflexion, inversion, and eversion in the D and N legs showed no significant differences at the beginning of the competition season and after 8 weeks of the competition season (*P* *≥* *0.05*). The ankle ROM values for active plantar flexion, inversion, and eversion were within the normal range; the exception was the ankle dorsiflexion ROM, which was lower at 10°—11° compared with the norm (15°—20° in the non-weight bearing positions) (60). The active ankle dorsiflexion range of movement decreased significantly with age in 7 to 17-year-old football players; no differences in ROM were detected between the dominant and non-dominant legs (69). Moreno-Pérez et al. (70) observed the ankle dorsiflexion ROM decrease from pre-season to mid-season (8.1% in the D, and 9.6% in the N leg) and post-season (13.8% in the D, and 12.5% in the N leg) in professional football players. Restricted ankle dorsiflexion ROM was observed in 30% of all players in post-season compared with pre-season. A certain degree of stiffness in the lower extremity is essential for effectively storing and reusing the elastic energy of connective tissue during stretch-shortening cycle activities: it enables greater concentric force output at push-off, higher running speed and rapid changes of direction (71). Therefore, a decrease in active ankle dorsiflexion ROM offers specific benefits for football players, as it is associated with increased foot rigidity (stiffness). Powerful kicks are achieved through high foot velocity and the rigidity of the foot and leg at impact (72). Nevertheless, decreased ankle dorsiflexion ROM contributes to the development of compensatory movement strategies, and dorsiflexion ROM positively correlates with dynamic balance performance; therefore, reduced ankle dorsiflexion ROM could increase the risk of falls and recurrence of sports injuries (73).

The passive plantar flexion ROM was significantly reduced in both ankles after 8 weeks of the competition season (*P* *≤* *0.04*). This may be attributed to greater ankle joint stiffness resulting from increased rigidity in the connective tissue structures of the feet, which, as reported by Loram & Lakie (26), helped improve static balance. These authors (26) revealed, using electromyography, that ankle stiffness, a determinant of static balance performance, cannot be regulated by the stretch reflex or the nervous system in quiet standing. This stiffness was determined by the foot, Achilles tendon, and aponeurosis rather than by the activated calf muscle fibers. These authors suggested that the triceps surae muscles maintained dynamic balance by predictively controlling the proximal offset of the spring-like element in a ballistic-like manner. Therefore, leg muscle strength should be a determinant of dynamic balance performance. Our data confirmed that static balance characteristics positively correlated with ankle ROM, while dynamic balance (TTD angle) negatively correlated with the peak torques of ankle dorsiflexion, eversion, and inversion. Our results were in good agreement with the findings of Kim & Kim (22), who observed that passive and active ankle plantar flexion ROM significantly positively correlated with body CoP sway length and speed of static balance performance in healthy young adults. The correlation was closer with the passive ROM (*R* *=* *0.431–0.525, P* *<* *0.01*) than with the active ROM (*R* *=* *0.274–0.325, P* *<* *0.05*). This allowed us to suggest that lower ankle joint stiffness (especially passive) is associated with greater ROM in plantar flexion and poorer static balance. The results of Kim & Kim (22) demonstrated that increased shin muscle strength led to improved static balance performance. These authors observed significant negative correlations between the isometric peak force of the ankle plantar and dorsal flexor muscles and the characteristics of static balance (body center of pressure CoP, sway length and velocity), with correlation coefficients ranging from −0.337 to −0.442 (*P* *<* *0.05*) in young, healthy adults. These results suggested that higher levels of static strength in the shin muscles were linked to better static balance.

At the beginning of the competition season and after the first 8 weeks spent in the competition season, the absolute isometric peak torques of ankle plantar flexion and dorsiflexion were higher in the D leg than in the N leg (*P* *≤* *0.045*). Nevertheless, relative isometric peak torque side-asymmetry was detected only for ankle dorsiflexion, where the relative peak torque was higher in the D leg compared with the N leg (*P* *≤* *0.039*). Significant side asymmetry of peak torques was not observed in the ankle invertor and evertor muscles (*P* *≥* *0.05*). Eight weeks of the competition season resulted in an increase only in absolute and relative ankle dorsiflexion peak torques (*P* *≤* *0.003*). Significant negative correlations were observed between relative ankle dorsiflexion torque, relative ankle eversion and inversion torques, and dynamic balance, characterized by the TTD angle (*R* *=* *−0.386, P* *=* *0.005; R* *=* *−0.337, P* *=* *0.015; R* *=* *−0.287, P* *=* *0.039*, respectively): the trunk sway angle reduced with an increase in ankle dorsal flexor, evertor, and invertor muscle strength. Chtara et al. (11) used the same dynamometer as we did in our study and found that only ankle dorsiflexor muscles were stronger in the D leg than in the N leg (*P* *=* *0.032*); the isometric peak force of the ankle plantar flexor, invertor, and evertor muscles did not differ between both legs (*P* *≥* *0.204*) in elite 16-year-old football players. They observed significant positive correlations between peak isometric strength of the ankle plantar flexors, dorsiflexors, and evertors and dynamic balance performance in the lower-limb Y-balance test (anterior and posteromedial reach distances) in young football players.

The kinesthetic sense acuity (the average track error ATE score) performed better on the test with the D leg than with the N leg (*P* *=* *0.021*) at the beginning and after the first 8 weeks of the competition season. The mean kinesthetic sense test trial time was shorter when performing the task with the N leg in both testing sessions (*P* *≤* *0.032*). Neither characteristic of the feet's kinesthetic sense acuity changed after 8 weeks spent in the competition period. The effectiveness of proprioceptive training was confirmed in the study by Gidu et al. (31): 14-year-old football players participated in an 8-week program (including 12 Bosu ball exercises to enhance balance, stability, and strength) with four 30 min sessions per week. The static balance (Balance Error Scoring System) score improved after this intervention, with 5.828 ± 1.017 fewer errors in the experimental group; in the control group, the reduction was only 0.780 ± 0.895 errors. No impact of spending many hours in training sessions and competitions on the feet's kinesthetic acuity was observed in our study. The average trace error (ATE) in our adolescent football players was considered normal but not optimal (with values ranging from 35% to 100%) (59). The explanation could be the lack of additional proprioceptive training in the program for our football players; at the same time, there was significant growth in the players (the mean height increase of 0.8 cm and body mass of 1.02 kg), which could lead to slower processing of motor commands in the adolescent's brain due to the rapid growth in legs' length, resulting in a worsening of kinesthetic sense acuity (36, 37). Furthermore, the kinesthetic accuracy assessment test on the ProKin 252 stabilometric platform was general and not specifically designed for football players. Although the test used in this study is not specific to football, we propose that its precision reflects the participants' capacity to learn new motor skills, because the movement sequence itself is not rehearsed as a sport-specific technique.

The study is limited by its small sample size and the homogeneity of the cohort, as all participants were drawn from a single club and trained under identical coaching conditions. The relatively small sample size may have limited the ability to detect a statistically significant impact of the training on specific balance outcomes and lower-limb characteristics in football players, increasing the risk of a Type II error. Differences in maturation rates between participants may also have influenced the results. The longitudinal observational design without a control group makes it difficult to disentangle training- and competition-related adaptations from growth- and maturation-related changes in this adolescent cohort. Some of the observed gains in stabilometric platform scores may be partially explained by a learning effect, since participants may have improved in executing the specific visuomotor task between the two testing sessions. Some uncontrollable factors could affect the results, such as participants' mood, sleep quality, stress level, and nutritional and health status.

The research hypothesis received only partial confirmation: during the first 8 weeks of the competition period (spring), static and dynamic balance improved in both the D and N leg stances, while peak torque gains were observed solely for dorsiflexion in both legs. In contrast, active ankle ROMs did not change, but the passive ROM of ankle plantar flexion reduced; kinesthetic sense acuity remained the same in the first 8 weeks of the competition season in adolescent football players.

In his review article, Wik (74) noted that at least one in three players sustained an injury over the football season, with the highest injury incidence observed in 15- to 16-year-old male players after their growth spurt. Maintaining good balance is essential for reducing falls and, consequently, lowering the likelihood of injuries in football players (75). Our observations indicated that an 8-week period of regular football training coincided with enhancements in (1) static balance, as evidenced by a reduction in MLS speed over the competitive phase (*η*^2^ *=* *0.131*), which represents a moderate-to-large effect, indicating a meaningful improvement in postural control; and (2) dynamic balance performance, as evidenced by the decrease in TTD angle, also showed a moderate-to-large effect (*η*^2^ *=* *0.128*), supporting the functional relevance of changes in dynamic balance even in growing adolescent players. These results are consistent with reports that 13-year-old male football players show superior static balance when compared with non-athletic boys of the same age (76).

Another important contributor to reducing the risk of falls and injuries is improved lower leg muscle strength (22, 23). We found significant associations between dynamic balance (TTD) and ankle dorsiflexion, inversion, and eversion relative peak torques (*P* ≤ 0.039), with the strongest correlation observed with the ankle dorsiflexion relative peak torque (*P* *≤* *0.005*). Consistent football practice was associated with a higher peak torque in ankle dorsiflexion, with this change representing a large effect (*η*^2^ *=* *0.328*), suggesting that neuromuscular adaptations contributing to enhanced dynamic balance occurred during the first 8 weeks of the competitive season.

We observed that ankle dorsiflexion ROM in the non-weight-bearing positions was lower (10°–11°) than the norm (15°–20°) (60). A decrease in ankle dorsiflexion ROM with age in growing footballers (69) and during the competition season in professional players (70) was likewise reported by other authors. This decrease in active ankle dorsiflexion ROM offers specific benefits for football players: when the foot becomes more rigid or stiff, it enables more forceful kicks, produced by the combination of high foot speed and a firm foot–leg position at the moment of impact (72). On the other hand, limited ankle dorsiflexion ROM constrains full knee flexion during weight-bearing exercises, leading to greater knee valgus movement and higher peak ground reaction forces during landing, squatting, and step-down tasks (77, 78). This indicates that limited dorsiflexion may diminish the ability to absorb forces, which could in turn increase the load on the musculoskeletal system at the ankle and knee due to compensatory movements in the sagittal and/or frontal planes (77, 78). Therefore, ankle dorsiflexion deficit is a risk factor for various lower-limb injuries (73, 79). Nevertheless, we suggest that other advantages of routine football training (improved static and dynamic balance and shin muscle strength) could compensate for the negative effect of reduced ankle dorsiflexion ROM in reducing the likelihood of falls and associated injuries. This is proved by the study of Rein et al. (80), who observed that, nevertheless, professional footballers exhibited substantially less right-foot dorsiflexion ROM, greater pronation in both feet, and reduced supination of the left foot in comparison to amateur players; there were no significant differences in balance control between the two groups.

Future research on static and dynamic balance performance and its determinants in young football players should include longer observation periods throughout the training macrocycle and involve larger, more diverse groups, including players from multiple clubs and age-matched controls. This approach will better clarify developmental patterns in balance, proprioception, and neuromuscular performance in youth football.

## 5 Conclusion

Our observations demonstrate that routine football training and competition was associated with favorable adaptations in balance and neuromuscular performance during the first 8 weeks of the competition period, such as static balance (reduction of the body CoP sway speed) and dynamic balance (decrease in the TTD angle) in one leg stance, peak ankle dorsiflexion torques enhancement and ankle joint stiffness increase (reduction in ankle passive plantar flexion ROM). However, they were insufficient to improve kinesthetic sense without dedicated proprioceptive training. Adolescent players reproduced active foot trajectories with a precision similar to that of typical individuals without prior training. We recommend adding at least two weekly 30 min sessions after the warm-up of the regular training routine, focused on balance and proprioceptive exercises performed on unstable surfaces, complemented by movement-control drills, to improve movement accuracy and reduce motor-control impairments during phases of rapid growth.

## Data Availability

The original contributions presented in the study are included in the article/supplementary material. Primary pseudonymised datasets are available on request to the corresponding author. The data are published in the RSU Dataverse domain (link: https://dataverse.rsu.lv), which has all the necessary measures to ensure secure long-term preservation and curation; the license used is Attribution-NonCommercial-ShareAlike 4.0.
