# Supplementary material for: Changes in balance performance and Its determining factors in the lower leg during the competition season in adolescent football players
Source: Front Sports Act Living. 2026 May 28;8:1769946. doi: 10.3389/fspor.2026.1769946 (PMC13253643; doi:10.3389/fspor.2026.1769946)
Supplement: Supplementary file 1 [file Table1.docx]

Supplement Table 1

The results of the normality tests - Shapiro–Wilk Test Results (Data Used for Statistical Analyses).

| **Variable** | **Time** | **Leg** | ***W*** | ***P-value*** | **Normality** |
| --- | --- | --- | --- | --- | --- |
| Height, cm | Pre |  | *0.842* | *0.022* | No |
|  | Post |  | *0.849* | *0.028* | No |
| Height (log) | Pre |  | *0.822* | *0.013* | No |
|  | Post |  | *0.831* | *0.016* | No |
| Weight, kg | Pre |  | *0.947* | *0.550* | Yes |
|  | Post |  | *0.952* | *0.626* | Yes |
| BMI, kg/m2 | Pre |  | *0.929* | *0.326* | Yes |
|  | Post |  | *0.916* | *0.220* | Yes |
| TSI | Pre | Dominant | *0.896* | *0.118* | Yes |
|  |  | Non-Dominant | *0.881* | *0.073* | Yes |
|  | Post | Dominant | *0.848* | *0.027* | No |
|  |  | Non-Dominant | *0.916* | *0.222* | Yes |
| TSI (log) | Pre | Dominant | *0.931* | *0.354* | Yes |
|  |  | Non-Dominant | *0.959* | *0.740* | Yes |
|  | Post | Dominant | *0.875* | *0.062* | Yes |
|  |  | Non-Dominant | *0.911* | *0.190* | Yes |
| Trunk total standard deviation, degrees | Pre | Dominant | *0.782* | *0.004* | No |
|  |  | Non-Dominant | *0.771* | *0.003* | No |
|  | Post | Dominant | *0.921* | *0.257* | Yes |
|  |  | Non-Dominant | *0.792* | *0.005* | No |
| Trunk total standard deviation (log) | Pre | Dominant | *0.954* | *0.664* | Yes |
|  |  | Non-Dominant | *0.924* | *0.283* | Yes |
|  | Post | Dominant | *0.923* | *0.274* | Yes |
|  |  | Non-Dominant | *0.870* | *0.061* | Yes |
| CoP Ellipse area, mm2 | Pre | Dominant | *0.926* | *0.305* | Yes |
|  |  | Non-Dominant | *0.970* | *0.896* | Yes |
|  | Post | Dominant | *0.882* | *0.076* | Yes |
|  |  | Non-Dominant | *0.898* | *0.125* | Yes |
| CoP Perimeter, mm | Pre | Dominant | *0.797* | *0.006* | No |
|  |  | Non-Dominant | *0.967* | *0.855* | Yes |
|  | Post | Dominant | *0.903* | *0.146* | Yes |
|  |  | Non-Dominant | *0.954* | *0.659* | Yes |
| CoP Perimeter (log) | Pre | Dominant | *0.531* | *<0.001* | No |
|  |  | Non-Dominant | *0.924* | *0.286* | Yes |
|  | Post | Dominant | *0.889* | *0.096* | Yes |
|  |  | Non-Dominant | *0.931* | *0.356* | Yes |
| CoP APS speed, mm/s | Pre | Dominant | *0.929* | *0.333* | Yes |
|  |  | Non-Dominant | *0.885* | *0.084* | Yes |
|  | Post | Dominant | *0.971* | *0.902* | Yes |
|  |  | Non-Dominant | *0.948* | *0.568* | Yes |
| CoP MLS speed, mm/s | Pre | Dominant | *0.934* | *0.383* | Yes |
|  |  | Non-Dominant | *0.945* | *0.524* | Yes |
|  | Post | Dominant | *0.962* | *0.786* | Yes |
|  |  | Non-Dominant | *0.931* | *0.350* | Yes |
| Peak Force Plantar flexors, N | Pre | Dominant | *0.882* | *0.076* | Yes |
|  |  | Non-Dominant | *0.892* | *0.104* | Yes |
|  | Post | Dominant | *0.895* | *0.115* | Yes |
|  |  | Non-Dominant | *0.952* | *0.634* | Yes |
| Peak Force Dorsiflexors, N | Pre | Dominant | *0.971* | *0.908* | Yes |
|  |  | Non-Dominant | *0.967* | *0.859* | Yes |
|  | Post | Dominant | *0.923* | *0.274* | Yes |
|  |  | Non-Dominant | *0.927* | *0.311* | Yes |
| Peak Force Invertors, N | Pre | Dominant | *0.867* | *0.048* | No |
|  |  | Non-Dominant | *0.968* | *0.875* | Yes |
|  | Post | Dominant | *0.927* | *0.310* | Yes |
|  |  | Non-Dominant | *0.887* | *0.089* | Yes |
| Peak Force Invertors (log) | Pre | Dominant | *0.784* | *0.004* | No |
|  |  | Non-Dominant | *0.897* | *0.123* | Yes |
|  | Post | Dominant | *0.936* | *0.410* | Yes |
|  |  | Non-Dominant | *0.904* | *0.152* | Yes |
| Peak Force Evertors, N | Pre | Dominant | *0.893* | *0.107* | Yes |
|  |  | Non-Dominant | *0.953* | *0.637* | Yes |
|  | Post | Dominant | *0.908* | *0.173* | Yes |
|  |  | Non-Dominant | *0.896* | *0.119* | Yes |
| Peak Torque Plantar flexion, Nm | Pre | Dominant | *0.883* | *0.078* | Yes |
|  |  | Non-Dominant | *0.920* | *0.250* | Yes |
|  | Post | Dominant | *0.960* | *0.759* | Yes |
|  |  | Non-Dominant | *0.963* | *0.806* | Yes |
| Peak Torque Dorsal flexion, Nm | Pre | Dominant | *0.886* | *0.087* | Yes |
|  |  | Non-Dominant | *0.963* | *0.806* | Yes |
|  | Post | Dominant | *0.901* | *0.140* | Yes |
|  |  | Non-Dominant | *0.906* | *0.159* | Yes |
| Peak Torque Inversion, Nm | Pre | Dominant | *0.890* | *0.097* | Yes |
|  |  | Non-Dominant | *0.941* | *0.470* | Yes |
|  | Post | Dominant | *0.860* | *0.038* | No |
|  |  | Non-Dominant | *0.869* | *0.051* | Yes |
| Peak Torque Inversion (log) | Pre | Dominant | *0.860* | *0.039* | No |
|  |  | Non-Dominant | *0.920* | *0.250* | Yes |
|  | Post | Dominant | *0.922* | *0.267* | Yes |
|  |  | Non-Dominant | *0.933* | *0.368* | Yes |
| Peak Torque Eversion, Nm | Pre | Dominant | *0.934* | *0.387* | Yes |
|  |  | Non-Dominant | *0.971* | *0.901* | Yes |
|  | Post | Dominant | *0.900* | *0.132* | Yes |
|  |  | Non-Dominant | *0.927* | *0.310* | Yes |
| Relative Peak Torque Plantar flexion, Nm/kg | Pre | Dominant | *0.836* | *0.019* | No |
|  |  | Non-Dominant | *0.919* | *0.240* | Yes |
|  | Post | Dominant | *0.917* | *0.227* | Yes |
|  |  | Non-Dominant | *0.834* | *0.018* | No |
| Relative Peak Torque Plantar flexion (log) | Pre | Dominant | *0.876* | *0.062* | Yes |
|  |  | Non-Dominant | *0.942* | *0.483* | Yes |
|  | Post | Dominant | *0.943* | *0.497* | Yes |
|  |  | Non-Dominant | *0.867* | *0.048* | No |
| Relative Peak Torque Dorsal flexion, Nm/kg | Pre | Dominant | *0.933* | *0.378* | Yes |
|  |  | Non-Dominant | *0.961* | *0.776* | Yes |
|  | Post | Dominant | *0.940* | *0.459* | Yes |
|  |  | Non-Dominant | *0.979* | *0.972* | Yes |
| Relative Peak Torque Inversion, Nm/kg | Pre | Dominant | *0.881* | *0.074* | Yes |
|  |  | Non-Dominant | *0.985* | *0.995* | Yes |
|  | Post | Dominant | *0.887* | *0.088* | Yes |
|  |  | Non-Dominant | *0.872* | *0.055* | Yes |
| Relative Peak Torque Eversion, Nm/kg | Pre | Dominant | *0.906* | *0.161* | Yes |
|  |  | Non-Dominant | *0.945* | *0.527* | Yes |
|  | Post | Dominant | *0.875* | *0.061* | Yes |
|  |  | Non-Dominant | *0.909* | *0.178* | Yes |
| Active ROM Plantar flexion, degrees | Pre | Dominant | *0.923* | *0.277* | Yes |
|  |  | Non-Dominant | *0.909* | *0.177* | Yes |
|  | Post | Dominant | *0.832* | *0.017* | No |
|  |  | Non-Dominant | *0.927* | *0.309* | Yes |
| Active ROM Plantar flexion (log) | Pre | Dominant | *0.906* | *0.162* | Yes |
|  |  | Non-Dominant | *0.886* | *0.086* | Yes |
|  | Post | Dominant | *0.772* | *0.003* | No |
|  |  | Non-Dominant | *0.908* | *0.174* | Yes |
| Active ROM Dorsal flexion, degrees | Pre | Dominant | *0.957* | *0.699* | Yes |
|  |  | Non-Dominant | *0.965* | *0.822* | Yes |
|  | Post | Dominant | *0.942* | *0.485* | Yes |
|  |  | Non-Dominant | *0.942* | *0.485* | Yes |
| Active ROM Inversion, degrees | Pre | Dominant | *0.888* | *0.092* | Yes |
|  |  | Non-Dominant | *0.900* | *0.132* | Yes |
|  | Post | Dominant | *0.935* | *0.095* | Yes |
|  |  | Non-Dominant | *0.830* | *0.016* | No |
| Active ROM Inversion (log) | Pre | Dominant | *0.883* | *0.078* | Yes |
|  |  | Non-Dominant | *0.904* | *0.153* | Yes |
|  | Post | Dominant | *0.948* | *0.569* | Yes |
|  |  | Non-Dominant | *0.865* | *0.045* | No |
| Active ROM Eversion, degrees | Pre | Dominant | *0.973* | *0.924* | Yes |
|  |  | Non-Dominant | *0.834* | *0.018* | No |
|  | Post | Dominant | *0.828* | *0.015* | No |
|  |  | Non-Dominant | *0.835* | *0.018* | No |
| Active ROM Eversion (log) | Pre | Dominant | *0.955* | *0.672* | Yes |
|  |  | Non-Dominant | *0.847* | *0.026* | No |
|  | Post | Dominant | *0.832* | *0.017* | No |
|  |  | Non-Dominant | *0.890* | *0.101* | Yes |
| Passive ROM Plantar flexion, degrees | Pre | Dominant | *0.952* | *0.625* | Yes |
|  |  | Non-Dominant | *0.789* | *0.005* | No |
|  | Post | Dominant | *0.905* | *0.157* | Yes |
|  |  | Non-Dominant | *0.926* | *0.297* | Yes |
| Passive ROM Plantar flexion (log) | Pre | Dominant | *0.929* | *0.331* | Yes |
|  |  | Non-Dominant | *0.722* | *0.001* | No |
|  | Post | Dominant | *0.861* | *0.041* | No |
|  |  | Non-Dominant | *0.920* | *0.251* | Yes |
| Passive ROM Dorsal flexion, degrees | Pre | Dominant | *0.973* | *0.922* | Yes |
|  |  | Non-Dominant | *0.984* | *0.994* | Yes |
|  | Post | Dominant | *0.916* | *0.224* | Yes |
|  |  | Non-Dominant | *0.937* | *0.423* | Yes |
| Passive ROM Inversion, degrees | Pre | Dominant | *0.933* | *0.370* | Yes |
|  |  | Non-Dominant | *0.942* | *0.485* | Yes |
|  | Post | Dominant | *0.936* | *0.411* | Yes |
|  |  | Non-Dominant | *0.886* | *0.086* | Yes |
| Passive ROM Eversion, degrees | Pre | Dominant | *0.968* | *0.873* | Yes |
|  |  | Non-Dominant | *0.965* | *0.803* | Yes |
|  | Post | Dominant | *0.956* | *0.691* | Yes |
|  |  | Non-Dominant | *0.951* | *0.615* | Yes |
| Average trace error ATE, % | Pre | Dominant | *0.944* | *0.514* | Yes |
|  |  | Non-Dominant | *0.939* | *0.442* | Yes |
|  | Post | Dominant | *0.948* | *0.575* | Yes |
|  |  | Non-Dominant | *0.909* | *0.175* | Yes |
| Kinesthetic acuity test trial time, s | Pre | Dominant | *0.983* | *0.991* | Yes |
|  |  | Non-Dominant | *0.853* | *0.031* | No |
|  | Post | Dominant | *0.790* | *0.005* | No |
|  |  | Non-Dominant | *0.855* | *0.033* | No |
| Kinesthetic acuity test trial time (log) | Pre | Dominant | *0.984* | *0.994* | Yes |
|  |  | Non-Dominant | *0.923* | *0.275* | Yes |
|  | Post | Dominant | *0.892* | *0.103* | Yes |
|  |  | Non-Dominant | *0.947* | *0.549* | Yes |
